# Amharic version of the work-life balance scale: validation and psychometric properties

**DOI:** 10.1186/s40359-025-03773-y

**Published:** 2025-12-06

**Authors:** Wondu Teshome, Daniel Tefera, Dawit Demlie, Hareg Teklu, Yitagesu Fikadu

**Affiliations:** 1 Department of Psychology, College of Social Science and Humanities, Dire Dawa University, Dire Dawa, Ethiopia; 2 School of Psychology, College of Education and Behavioral Studies, Addis Ababa University, Addis Ababa, Ethiopia; 3 Department of Sociology, College of Social Science and Humanities, Dire Dawa University, Dire Dawa, Ethiopia

**Keywords:** Work-life balance scale, Psychometric properties, Validation, Health professionals

## Abstract

**Background:**

Work-life balance scale needs to be validated and adapted across various profession and socio-cultural contexts.The aim of this study was to assess the factor structures and psychometric properties of the work-life balance scale for health professionals working in hospitals in the Eastern Ethiopian context.

**Methods:**

A sample of 338 health professionals (of which 50.6% were female and 49.4% were male) from three public and three private hospitals were selected through simple random and stratified proportional sampling techniques.

**Results:**

The number of factors was determined using maximum likelihood with the promax rotation method and exploratory factor analysis, where four factors were extracted from the work-life balance scale, namely ‘personal life enhancement of work, ‘work enhancement of personal life, ‘personal life interference with work’, and ‘work interference with personal life. Moreover, confirmatory factor analysis, descriptive statistics, validity and reliability analysis were employed as methods of data analysis. In conclusion, the work-life balance scale showed strong psychometric properties and can be used as reliable instruments to assess and measure the work-life balance of health professionals.

**Conclusion:**

The Amharic version of work-life balance scale proves valid and reliable tool for health professionals working in hospitals in Eastern Ethiopia. However, it requires further culturally appropriate revision through involving other large samples of health professionals across all parts of Ethiopia.

## Background

Work, family, and personal life are the most important aspects of human life. The balance between work and non-work affairs has been a growing issues globally among health professionals. In fact, balancing work and non-work affairs is the most challenging thing for health professionals because of their multiple duties and responsibilities related to their work, family, and personal lives. Researchers studied more widely the linkage between work and family domains and indicated that the intersection of work and family roles has become recognized as an important area of study within industrial and organizational psychology [1, 2].

Several past studies have been done on areas of work-family interface [3–6]. However, only a few studies attemptted to look at the linkages between work, family, and personal life aspects and investigated the person-work-family interface in their studies [7–10]. In this regard [2], argued that researchers have criticized the “work-family” framework as too narrow in that it neglects other life roles important to individuals (p. 699) [1]. defined the concept of work-life balance (WLB) as “referring to work as paid employment, life as the activities outside work, and balance as the equal weight given to both work and non-work activities” (p. 262). To different scholars, the term WLB means different things. For instance [11], described that there is no single understanding or use of the term WLB; rather, there are multiple and overlapping WLB discourses within organizations and among academic researchers that need further conceptualization.

Health professionals are always striving hard to save lives, as they are front-liners in dealing with patient care services. Previous researchers [12–14] defined the term WLB as the compatibility, harmony, and equilibrium between professional work and non-work affairs. Most researchers developed WLB measurement tools as constituting aspects of work and personal life interference and enhancement [15–18]. Various studies also similarly argued that WLB consists of four factors: work interference with personal life (WIPL), personal life interference with work (PLIW), work enhancement of personal life (WEPL), and personal life enhancement of work (PLEW) [19, 20].

According to [21], high levels of WLB are more positively associated with job and life satisfaction for individuals in individualistic cultures compared with individuals in collectivistic cultures. However, there are variations in empirical studies undertaken regarding the WLB scale from profession to profession and from context to context. That is why we plan to adapt, validate, and translate the English-version WLB tool to Amharic-version to fit our collective cultural context.

There have been several studies conducted to assess the WLB tool among large-scale industrial company employees. For instance [22], developed and standardized the WLB scale for the insurance sector employees in India and found that six factors of WLB scale, such as nature of work, work flexibility, workload, compensation, organizational support, and personal life, which were more than 0.7 Cronbach’s alpha coefficients. However, the tool developed for target groups such as industrial workers, bank and insurance workers, teachers, and hotel industry workers are different from health professionals working in a hospital context. Most tools developed and validated to investigate the issues of WLB among employees in the Western context.

To the best knowledge of the researchers, previously there were no standardized tools developed in the areas of WLB for health professionals working in African and Ethiopian contexts. Therefore, considering these gaps and limitations regarding the cross-cultural adaptation of the WLB scale, we try to redesign and adapt the WLB scale developed by [19] in the American context. By conducting content validity analysis, we modified some items and added a few items by consulting professional experts and conducting critical literature reviews. By considering the contexts of health professionals working in both public and private hospitals in Eastern Ethiopia, there is a need for new and modified instruments regarding WLB.

The development of valid and reliable WLB measurement instruments is essential to examine the cross-cultural variation of the tool. Previously, the WLB scale was translated into various languages such as French, Germany, Arabic, Chinese, Turkish, Indian etc. worldwide. The translation of the WLB scale into different languages indicated that the psychometric property differences of the scale from culture to culture, society to society, and context to context.

To date, there are limited studies that try to incorporate the issues of work-personal life interference and work-personal life enhancement into their studies of WLB tool. Hence, it is crucial to fill such gaps and limitations by investigating the psychometric properties of WLB of health professionals in public and private hospitals. However, there is a scarcity of studies conducted regarding WLB tools among health professionals in the Ethiopian context. Therefore, it is very essential to undertake this study to examine the validity of psychometric properties of WLB among health professionals from social psychological perspectives in the collective cultural context of Eastern Ethiopia.

Therefore, the researchers of the present study took the initiative to validate and adapt the Amharic version questionnaire so that it could be usable in the Ethiopian context by testing its psychometric properties and factor structures. This study aimed to test, validate, and analyze the psychometric properties of an Amharic version of the WLB scale for health professionals working in public and private hospitals in Eastern Ethiopia. The cross-cultural reliability and validity of WLB scale need to be investigated. Accordingly, the main objective of the present study was to examine the psychometric properties of the WLB scale for health professionals working in public and private hospitals in the Eastern Ethiopian context. Specifically, the study was intended to; (1) to explore the factors and structures of the Amharic-version of the WLB scale for health professionals. (2) to examine the reliability and validity of the Amharic-version of the WLB scale for health professionals, and (3) to investigate the cultural adaptation and validation of the psychometric properties of the Amharic-version of the WLB scale in Eastern Ethiopian context.

## Methods

### Design and study population

In this study, a cross-sectional design with quantitative research approach was used because the researchers collected data from health professionals in each public and private hospital at one given time. This study was conducted in both public and private hospitals found in Dire Dawa City Administration (DDCA) and Harari Regional State (HRS). According to the Dire Dawa Regional Health Bureau and human resource departments of each hospital, there are 795 health professionals in four public and five private hospitals in DDCA. According to the Harari Regional Health Bureau and human resource departments of each hospital, there are 727 health professionals in three public and two private hospitals.

### Inclusion and exclusion criteria

Inclusion criteria: Physicians, nurses, and midwives currently working in the selected hospitals during the data collection period. Exclusion criteria: Health professionals on annual leave, study leave, sick leave, or training at the time of data collection, and those not in clinical roles (e.g., administrative staff, or supportive staff of the hospitals).

### Sample and sampling technique

The study sample was taken from three public and three private hospitals in DDCA and HRS. According to [23], sample size depends on the objective of the study, the design of the study, the plan for statistical analysis, the degree of precision required for generalization, and the degree of confidence. According to [24], stratified random sampling is useful method for data collection if the population is heterogeneous and the entire heterogeneous population is divided in to a number of homogeneous groups, usually known as Strata, each of these groups is homogeneous within itself, and then units are sampled at random from each of these stratums [25]. provides a simplified formula to calculate sample sizes. This formula was used to calculate the sample sizes if the population is heterogeneous.

In this study, health professionals such as physician, nurse, and midwife varied from region to region, hospital to hospital in their work-life balance, job satisfaction, and job performance, to obtain a representative sample size, based on the above arguments [25], sample size formula was used.

According to [23], stratified sampling is employed if the population from which a sample is to be drawn does not constitute a homogeneous group. A stratified sampling technique is generally applied to obtain a representative sample. Under stratified sampling, the population is divided into several sub-populations that are individually more homogeneous than the total population (the different sub-populations are called ‘strata’) and then we select sample from each stratum to constitute a sample [23].

Therefore, based on the above ground, the sampling frame was constructed for each hospital separately by taking the health professionals’ list from the respective human resource management department of each hospital. Stratified proportional random sampling technique was applied to select the sample. Then, the study participants were selected from each stratum through simple random sampling technique. Hence, a total of 347 study participants were selected based on stratified and simple random sampling techniques. Relying on the heterogeneity effects arise from regional differences, types of hospital, and job title of health professionals, it is vital to form sub-population based on three strata formed as regions (DDCA and HRS), types of hospitals (public and private), and job titles (physician, nurse, and midwife).

From each stratum of region, hospital type, and job title, the participants were selected proportionally. Finally, based on the list of health professionals’ obtained from each public and private hospital, a sample of 347 study participants were selected based on simple random sampling technique. However, from the total samples of 347 participants who participated in this study, only 338 participants accurately filled out the questionnaire which accounted for 97% of the response rate.

### Procedure of data collection

The procedures of data collection started with training of the data collectors. Official letters of cooperation were given to all public and private hospitals from school of psychology in Addis Ababa University via prospective researcher to gain permission for data collection. Finally, the researcher asked the willingness of the selected health professionals’ to participate in the study and starts to collect the data via the questionnaires prepared for this purpose. A self-report Amharic version questionnaire was used to collect data from health professionals. The data was collected from 338 health professionals from six public and private hospitals. The data collection activities were conducted during March 10 to May 25, 2024.

### Tool adaptation, validation, and translation process

An expert panel consisting of subject matter experts consisted of eight volunteer professionals, four from Social Psychology, two each from Developmental Psychology, and Counseling Psychology and bilingual translators participated in the content validation process of the WLB tool. Content validity was then assessed using [26] content validity ratio formula. For this purpose, these expert judges were asked to rate each item as ‘Essential, Useful but not essential, or Not necessary’ in line with the definitions given for each scale and subscales.

Eight panels of experts with more than 10 years of experience in psychology fields evaluated the tool for content validity. Then, four language and subject matter experts conducted both forward and backward translations of the tool. Finally, four health professionals working in public hospitals rated the face’s validity, independently assessing its readability, appropriateness, clarity, and ambiguity. Their discussions helped resolve any inconsistencies and finalize the scale for the pretest. The English language experts were established equivalence between the original tools with backward translated English tool. They also ensured equivalence between the source and target versions and examined the questionnaires for any discrepancies.

The psychometric instrument properties were evaluated based on the following strategies and methods. The strategies involved in the psychometric instrument evaluations relied on content validity ratio (CVR), item-content validity index (I-CVI), and scale-content validity index (S-CVI). The above assessment was conducted based on the ratings provided by eight subject area experts who currently working in Addis Ababa and Dire Dawa universities.

According to [27], the recommended number of translators is a minimum of two forward translators to translate the instrument from the original language (source language) to the target language. Firstly, we developed a new Amharic version of WLB scale using the back translation procedure. Specifically, two Amharic native speakers translated the English version of WLB scale into Amharic language independently. The two translations were then compared, and no substantial differences were found. A bilingual speaker familiar with the psychological topic then back-translated the first final version into English. A minor revision was required after comparing the back-translation with the original version. Next, four health professionals working in public hospitals rated the face’s validity, independently assessing its readability, appropriateness, clarity, and ambiguity. Their discussions helped resolve any inconsistencies and finalize the scale for the pretest. The English language experts were established equivalence between the original tools with backward translated English tool. They also ensured equivalence between the source and target versions and examined the questionnaires for any discrepancies.

The equivalence between the original the English version of the tools and the backward translated version of the English language tools was made by subject matter experts and language experts. After providing them with an explanation about study purposes and the questionnaire, they were requested to evaluate the Amharic version instrument in terms of whether it indicated readability, clarity, appropriateness, the correct spelling of words, and freedom from ambiguity of items in the questionnaire.

To examine the validity of the pre-final Amharic version of the scales, cognitive debriefing was done with 15 Amharic speaking nurses randomly selected from France Emergency Hospital in Dire Dawa City Administration. The sample size for this cognitive debriefing pilot test was determined based on [27] suggestion that 10–40 individuals are enough for instrument translation pilot testing. Hence, respondents of the pilot tests were asked to rate the clarity of the items, instructions and response formats of the scales. They were also asked to offer their comments on making the language clearer and easier. Finally, the results from 15 nurses indicated that the inter-rater agreement among the sample was 95%. Therefore, the data collected from 338 health professionals in this study through the Amharic version of work-life balance scale.

#### Ethical consideration

The study protocol was reviewed and approved by the Research Ethics Committee of Addis Ababa University, School of Psychology (Ref.No.SoP.CEBS.01/24). All procedures are in accordance with the ethical standards of the Addis Ababa University, School of Psychology, Research Ethics Committee. The participants completed the informed consent form to participate in the study. This study was methodically and ethically approved by the research ethics committee of the School of Psychology in Addis Ababa University.

### Methods of data analysis

The methods of data analyses in this study were both descriptive and inferential statistics. At the first stage, statistical analyses assumptions checking and data screening was conducted on the univariate and multivariate levels. Preliminary analyses were conducted to ensure no violation of the assumptions of normality, linearity, multicollinearity, and homoscedasticity. As such, the normality, linearity and homoscedasticity, singularity and multivariate outliers were checked in the data screening stage. After proving all the aforementioned issues before enter into the main analysis, then the analysis process were conducted via the following consecutive steps:

First, we were conducted the content validity analysis on WLB scale. Second, we adapted the tools to the Ethiopian context and then all study variables’ descriptive statistics was computed. Thirdly, the exploratory factor analysis (EFA) on WLB scale was conducted via maximum likelihood (ML) model with promax rotation methods. Finally, confirmatory factor analysis (CFA) was performed for individual scales to confirm the measurement model. Data analysis methods were conducted via the statistical package for social sciences (SPSS-version-23) and analysis of moment structure (AMOS-version-23).

## Results

### Socio-demographic characteristics of the participants

The sample consisted of 338 participants selected from three public hospitals and three private hospitals in Eastern Ethiopia. The sample was selected from Dire Dawa City Administration (DDCA) and Harari Regional State (HRS). This study was conducted in public and private hospitals in Eastern Ethiopia, which comprises the DDCA and HRS. The two bigger and major cities in Eastern Ethiopia, namely Dire Dawa and Harar are selected because these areas are centers for all patients who come from both the Eastern and Western Hararghe highlands and the Somali region for their advanced referral and specialized public and private hospitals.

DDCA is located in the Eastern part of Ethiopia, at a distance of 515 km from Addis Ababa, the capital city of Ethiopia. DDA covers a total area of 1,977 km2, and it is divided into nine urban Kebeles and 38 farmers’ associations. HRS is located in the eastern part of Ethiopia, at a distance of 526 km from Addis Ababa. HRS is one of the regional states in Ethiopia, and it covers a total area of 311.25 km^2^. HRS has nine districts (six urban and three rural) and 36 kebeles.

The socio-demographic characteristics of the respondents including their age, gender, marital status, job title, type of hospital, name of hospital, and region. The participants age ranging from 20 to 66 years with the mean value of (M = 31.17, SD = 8.732). The participants gender composition indicated that 167 (49.4%) of the sample were males and 171 (50.6%) of the sample were females (Table 1).

**Table 1:** Socio-demographic characteristics of the participants’

Variables	Categories	Frequency	Percent
Gender	Male	167	49.4%
	Female	171	50.6%
Marital Status	Single	141	41.7%
	Married	197	58.3%
Job Title	Physician	67	19.8%
	Nurse	213	63.0%
	Midwife	58	17.2%
Type of Hospital	Public	285	84.3%
	Private	53	15.7%
Name of Hospital	Dilchora Referral Hospital	92	27.2%
	Sabian General Hospital	52	15.2%
	Yemariamwork Hospital	21	6.2%
	Iftu General Hospital	15	4.4%
	Haramaya University Hiwot Fana Comprehensive Specialized Hospital	142	42.0%
	Harar General Hospital	16	4.7%
Region	Dire Dawa City Administration	180	53.3%
	Harari Regional State	158	46.7%

As shown in Table 2, the participants marital status indicated that 197 (58%) of the participants were married and 141 (41%) of the participants were single. The majority of the participants 213 (67%) were nurses, 67 (19%) were physicians, and 58 (17%) were midwives in their job title. The majority of the participants 285 (84%) were from public hospitals and 53 (15%) were from private hospitals. Most of the health professionals 142 (42%) were selected from Haramaya University Hiwot Fana Comprehensive Specialized Hospital, 92 (27%) were selected from Dilchora Referral Hospital, and 52 (15%) were selected from Sabian General Hospital. The majority of the participants 180 (53%) were from Dire Dawa City Administration (DDCA) and 158 (46%) were from Harari Regional State (HRS).

**Table 2:** Items and reliability coefficients of the four work-life balance subscales

Work-Life Balance Subscales	No. of Items	Reliability Coefficient
Work Interference with Personal Life	4	0.933
Personal Life Interference with Work	5	0.867
Work Enhancement of Personal Life	5	0.884
Personal Life Enhancement of Work	5	0.884

### Reliability and validity of the instrument

#### Reliability of the instrument

According to [28], reliability is defined as the consistency or repeatability of an instrument. The most important form of reliability for multi-item instruments is the instrument’s internal consistency, which is the degree to which sets of items on an instrument behave in the same way. A scale’s internal consistency is quantified by a Cronbach’s alpha (α) value that ranges between 0 and 1, with optimal values ranging between 0.7 and 0.9 [28].

 Petscher et al. [29] and Watkins [30] revealed that reliability coefficients in the.90s are excellent and likely sufficient for clinical decisions, coefficients in the.80s are good and sufficient for non-critical decisions, coefficients in the.70s are adequate for group experimental research, and coefficients less than 0.70 are inadequate and potentially problematic. Analyses of the reliability estimates of the scales were computed by using the Cronbach alpha coefficient. Inter-item correlation on the correlation matrix among WLB subscales was examined. Finally, items from these standardized tools were used for further analysis in the Ethiopian public and private hospital context.

As shown in Table 2, the reliability estimate for work interference with personal life, personal life interference with work, work enhancement of personal life, and personal life enhancement of work subscales were 0.933, 0.867, 0.884, and 0.884 respectively. In this validation study, the reliability or internal consistency value for the over all 19-items of WLB was 0.882 Cronbach alpha coefficient.

### Content validity analysis

According to [26], two main computations are needed for content validity analysis. These are the content validity ratio (CVR) and content validity index (CVI). The content validity ratio (CVR) is an item statistic that is useful in the rejection or retention of specific items. After items have been identified for inclusion in the final form, the content validity index (CVI) is computed for the whole test. The CVI is simply the mean of the CVR values of the retained items [26]. On the other hand [31], reported that the formula for computation of CVR = (Ne – N/2)/(N/2) in which Ne is the number of panelists indicating “essential” and N is the total number of panelists. According to [31], the content validity ratio (CVR) for the items reflected the percentage of panelists rating an item as “essential.” Furthermore, CVI is extensively used by researchers for determining the content validity.

 Yusoff [32] reported the following major steps to calculate validity index: Item-level content validity index (I-CVI): the expert in agreement divided by the number of experts. Scale-Level Content Validity Index (S-CVI) based on the average of I-CVI scores across all items and divided by the number of items. Scale-Level Content Validity Index (S-CVI) based on the average of proportion relevance scores across all experts. Scale-Level Content Validity Index (S-CVI/UA) based on the universal agreement method, the average of UA scores across all items.

The content validity of the questionnaire indicated a higher level of CVR, I-CVI, and S-CVI, which were indicated by most researchers as having excellent levels of validity. The result of the overall average CVI for 21 items of the WLB scale was 0.964. The prior researchers reported that CVR and CVI for individual items and the overall S-CVI were used to determine the relevance of the psychometric tool. According to [33], a content validity index of ≥ 0.78 for individual items and ≥ 0.90 for the overall scale were considered acceptable.

The estimate of CVI ranges from 0 to 1. The acceptable cut-off value for CVI is based on the number of experts. According to [32], for six to eight experts, a CVI of at least 0.83 and above should be treated as relevant [34]. recommend that for a scale to be judged as having excellent content validity, it would be composed of items that had I-CVIs of 0.78 or higher and an S-CVI/Ave of 0.90 or higher. In this study context, the minimum value of CVR was 0.75, CVI was 0.875, and S-CVI was 0.964, as reported in this study.

During content analysis assessment, we included two items on each of the work/personal life enhancement dimensions. According to Bollen (1989), as cited in [19], the best practices in psychological measurement recommend the use of at least three items. Therefore, we were added two items on each work enhancement of personal life subscale and personal life enhancement of work subscale which previously only three items dimensions developed by [19]. Finally, 21-items (17 items adapted from [19] and four items newly developed items) were prepared for final data collection process.

### Convergent and discrminant validity

In this study, the values of composite reliability (CR) and the average variance extracted (AVE) of all WLB subscales were ≥ 0.86 and ≥ 0.57 respectively, and the value of the composite reliability of each WLB subscales was greater than the value of average variance extracted. Furthermore, the ***β*** -values for the factor loadings of all items in each WLB subscale were found to be ≥ 0.50. With regard to discriminant validity, the analysis revealed that the values of average variance extracted from each WLB subscale were greater than both the values of maximum shared squared variance and average shared squared variance.

The estimates from the four-factor structural solution yielded statistically significant and positively moderate relationships among the dimensions of WLB scale (the correlation coefficients between 0.31 and 0.35). The relationship between work interference with personal life (WIPL) and personal life interference with work (PLIW) is positive (*r* =.31) and the relationship between work enhancement of personal life (WEPL) and personal life enhancement of work (PLEW) is also positive (*r* =.35).

The average variance extracted (AVE) was assessed to determine convergent validity of each dimension which yielded the values that ranged between 0.58 and 0.76, which indicated that each dimension has above 0.50 AVE showing better convergent validity across work-life balance dimensions [35–37]. Furthermore, the convergent validity and discriminant validity of the measurement scale were assessed to check the validity of items in explaining their purported construct. The result revealed that the work-life balance scale has no validity concerns.

The work-life balance construct had a good convergent validity since its average variance extracted (AVE = 0.58, 0.59, 0.60, and 0.76), which indicated that all the four dimensions have more than the threshold value of 0.50 [37]. This construct had also a good discriminant validity since its AVE value (0.58) was greater than the maximum shred variance value (MSV = 0.01), showing higher discriminant validity [37]. Likewise, the construct of work-life balance had excellent discriminant validity descriptions since its AVE value (0.58) was greater than the MSV value (0.01). The maximum reliability (MaxR(H) of work-life balance constructs range from (0.92) for PLIW to (0.98) for PLEW. Table 3 presents the convergent and discriminant validity measures of the work-life balance scale.

**Table 3:** Convergent and discriminant validity of WLB sub-constructs

Constructs	CR	AVE	MSV	MaxR(H)	PLEW	WEPL	PLIW	WIPL
PLEW	0.929	0.769	0.060	0.989	0.877			
WEPL	0.872	0.593	0.060	0.971	0.246	0.770		
PLIW	0.874	0.589	0.140	0.925	−0.001	−0.098	0.767	
WIPL	0.882	0.609	0.140	0.926	0.375	−0.021	0.055	0.780

*CR* Composite Reliability, *AVE* Average Variance Extracted, *MSV* Maximum Shared Variance, *MaxR(H)* Maximum Reliability

### Descriptive statistics of the study variables

As a first step in data analysis process, the means, standard deviations, and inter-correlations of the study variables were computed. The item values labeled for the work-life balance scale ranged from a minimum of 1.00 to a maximum of 5.00. The mean and standard deviation score for the work-life balance scale ranged from *(M = 2.76*,* SD = 1.20)* for item PLIW3 to the higher mean *(M = 3.35*,* SD = 1.233)* for item PLEW3. Table 4 provides the item statistics of the work-life balance scale.

**Table 4:** Means, standard deviations, and Cronbach alpha results of the work-life balance scale

Items	Mean	SD	Corrected Item-Total Correlation	Cronbach’s Alpha if Item Deleted
WIPL2	3.06	1.281	0.432	0.810
WIPL3	3.20	1.136	0.341	0.815
WIPL4	2.97	1.276	0.382	0.813
WIPL5	3.06	1.271	0.427	0.810
PLIW1	2.86	1.291	0.305	0.817
PLIW2	2.86	1.287	0.382	0.813
PLIW3	2.76	1.204	0.401	0.812
PLIW4	2.84	1.304	0.438	0.810
PLIW6	2.91	1.331	0.388	0.812
WEPL1	3.31	1.171	0.360	0.814
WEPL2	3.14	1.230	0.446	0.809
WEPL3	3.19	1.131	0.353	0.814
WEPL4	3.19	1.186	0.450	0.809
WEPL5	3.07	1.163	0.443	0.810
PLEW1	3.30	1.165	0.413	0.811
PLEW2	3.22	1.279	0.397	0.812
PLEW3	3.35	1.233	0.315	0.816
PLEW4	3.31	1.236	0.483	0.807
PLEW5	3.30	1.291	0.386	0.812

As shown in Table 4, the item-total correlation coefficients of the WLB scale ranged from a minimum of 0.305 to a maximum of 0.483. In this WLB scale, item WIPL2, item WIPL5, item PLIW3, item PLIW4, item WEPL2, item WEPL4, item WEPL5, item PLEW1, and item PLEW4 have item-total correlation coefficient above 0.40.

As shown in Table 5, the mean and standard deviation score for (WLB) scale was (*M = 12.31*,* SD = 4.538)* for WIPL, for (PLIW) subscale was *(M = 14.25*,* SD = 5.187)*, for (WEPL) subscale was *(M = 15.92*,* SD = 4.866)*, and for (PLEW) subscale was *(M = 16.50*,* SD = 5.133).* The distribution of the score reported on the independent and dependent variables of the study is represented normally distributed types of data. According to [38], the data is normally distributed the skewness and kurtosis should be fall within the range of −2 and 2. As can be seen from Table 5, the result is indicated within the range; hence, the data is normally distributed.

**Table 5:** The descriptive statistics of the study variables

Variables	*N*	Mean	SD	Skewness	Kurtosis
WIPL	338	12.31	4.538	− 0.173	−1.025
PLIW	338	14.25	5.187	0.128	− 0.953
WEPL	338	15.92	4.866	− 0.358	− 0.716
PLEW	338	16.50	5.133	− 0.250	− 0.949

*WIPL* Work Interference with Personal Life, *PLIW* Personal Life Interference with Work, *WEPL* Work Enhancement of Personal Life, *PLEW* Personal Life Enhancement of Work

### Exploratory factor analysis results of WLB scale

To examine and test the stability of factor structure and consistence from population to population, from sample to sample, the researcher plan to check the factors are meaningfully correlated and represented by all newly adapted and translated items. To know the pattern of the items without imposing a priori constraints, conducting both exploratory factor analysis (EFA) and confirmatory factor analysis (CFA) would be enable me to first explore the pattern and structure of both the newly added items and adapted items freely EFA and then test the proposed model structure CFA to provide robust test of the factor structure and invariance despite items are adapted, translated, and added to measure the constructs in a different setting and sample.

According to [39], EFA is a data-driven approach such that no specifications are made in regard to the number of latent factors or to the pattern of relationships between the common factors and the indicators. In contrast, CFA is a type of structural equation modeling (SEM) that deals specifically with measurement models, that is, the relationships between observed measures or indicators.

In this study, EFA was conducted using maximum likelihood estimation (ML) method followed by promax rotation, which yielded four-factor solution to measure the Amharic version of work-life balance scale for health professionals using 19 observed indicators. The data were tested for sample adequacy and sphericity using the Kaiser-Meyer Olkin and Bartlett tests, and factor analysis supported the four-component solution.

This study assessed the adequacy of the recruited sample and the existence of significant relationships using Kaiser–Meyer–Olkin’s (KMO) test and Bartlett’s test of Sphericity, respectively. The KMO test value greater than 0.5 shows adequacy of the study sample size, and a significant Bartlett’s test (*p* <.05) indicates that correlation matrix has significant correlations among some variables [36]. The KMO’s value for sampling adequacy was 0.801 (acceptable: >0.50), and Bartlett’s Test of Sphericity test yielded significant chi-square value of 5005.548 with 171 degrees of freedom, which showed suitability of data for factor analysis [36, 38].

As shown in Table 6, the results from EFA revealed that WIPL, PLIW, WEPL, and PLEW explained 24.80%, 22.89, 13.89%, and 10.35% of variance on work-life balance of health professionals, respectively, which altogether accounted for 71.96% total variance explained by these four work-life balance constructs.

**Table 6:** Total variance explained by EFA results for WLB scale

Factor	Initial Eigenvalues			Extraction Sums of Squared Loadings			Rotation Sums of Squared Loading
	Total	% of Variance	Cumulative %	Total	% of Variance	Cumulative %	Total
1	4.713	24.805	24.805	3.548	18.672	18.672	3.433
2	4.350	22.897	47.702	2.765	14.552	33.223	3.612
3	2.641	13.899	61.601	4.244	22.339	55.562	3.590
4	1.968	10.359	71.960	1.796	9.450	65.012	3.331

Extraction Method: Maximum Likelihood Model with Promax Rotation

The eigenvalues ranged between 1.96 for PLEW and 4.71 for WIPL. The overall factor loadings in EFA ranged from 0.495 to 0.992. During the process of EFA, among the total 21 items two items (WIPL1) and (PLIW5) which had 0.184 and 0.199 communalities were deleted and 19-items which had above 0.40 factor loadings were retained for final factor analysis with four dimension of work-life balance constructs.

As shown in Table 7, the results of EFA revealed that the factor loadings on the four dimensions namely WIPL, PLEW, WEPL, and PLIW of WLB scale with the total of 19-items. Four factors were identified through maximum likelihood factor extraction and promax rotation method. Accordingly, 19 (nineteen) items were loaded in four factors with significant and positive loading values. Hence, items number WIPL2, WIPL5, WIPL4, and WIPL3 were loaded on Factor I; items number PLEW4, PLEW5, PLEW1, PLEW2, and PLEW3 were significantly loaded on Factor II; items number WEPL4, WEPL5, WEPL2, WEPL3, and WEPL1, were significantly loaded on Factor III; and items number PLIW2, PLIW1, PLIW6, PLIW3, and PLIW4 were significantly loaded on Factor IV.

**Table 7:**
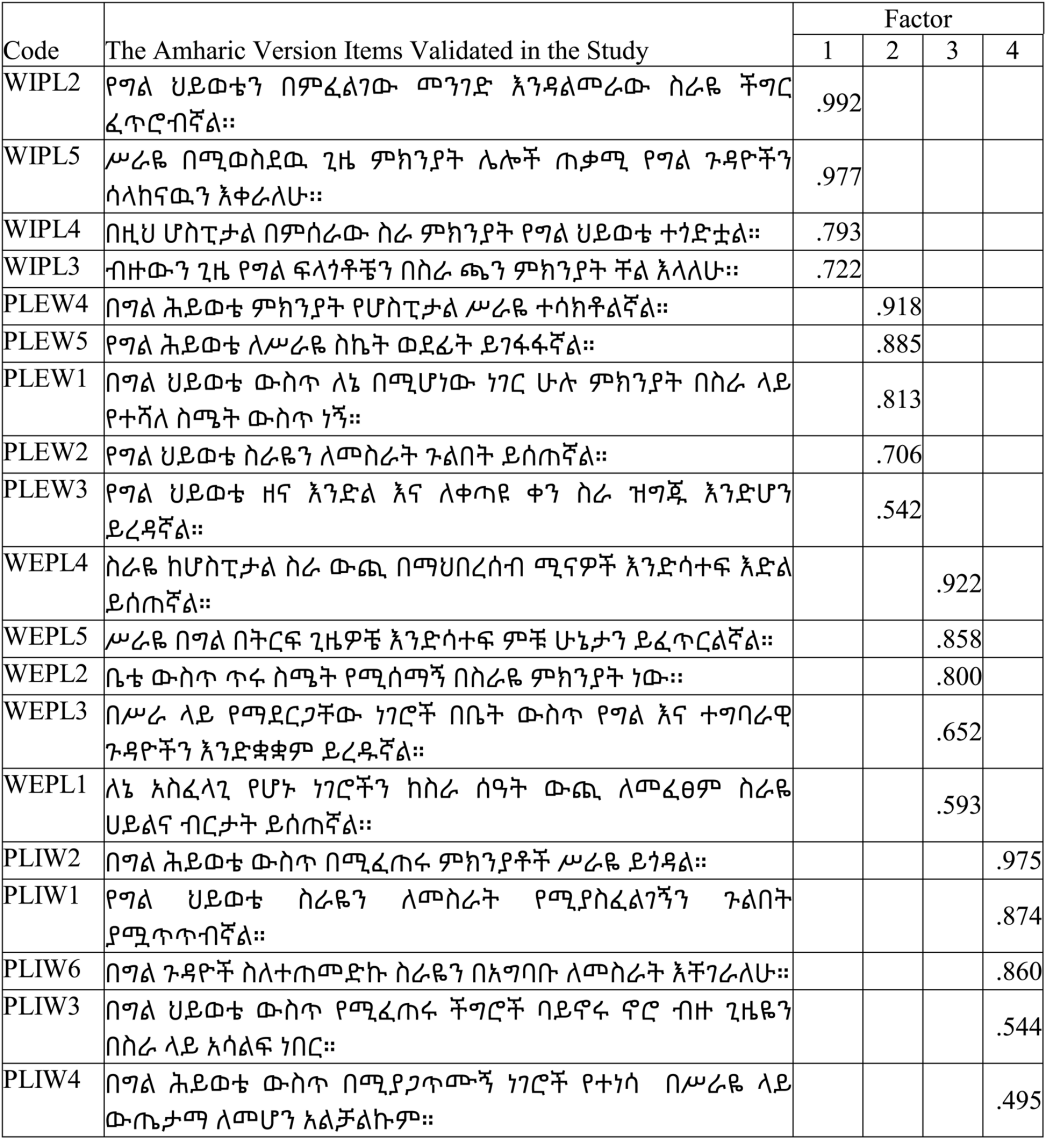
Factor loading on the four dimensions of WLB scale amharic version

Extraction Method: Maximum Likelihood. Rotation Method: Promax

As it can be observed from factor loading and rotation table, item number PLIW4 has lower loading values. Though this item has low loading values compared to other items, it was not deleted because it has nearly consistent and it’s extraction values and loading value was > 0.4. Lastly, it has been recognized that Factor I, Factor II, Factor III, and Factor IV represent the four components of WLB scale. These components are Work Interference with Personal Life with 4 (four) items: item number WIPL2, WIPL5, WIPL4, and WIPL3; Personal Life Enhancement of Work with five (5) items: Item number PLEW4, PLEW5, PLEW1, PLEW2, and PLEW3; Work Enhancement of Personal Life with five (5) items: Item number WEPL4, WEPL5, WEPL2, WEPL3, and WEPL1; and Personal Life Interference with Work subscale which consists of five items: item number PLIW2, PLIW1, PLIW6, PLIW3, and PLIW4.

**Fig. 1:**
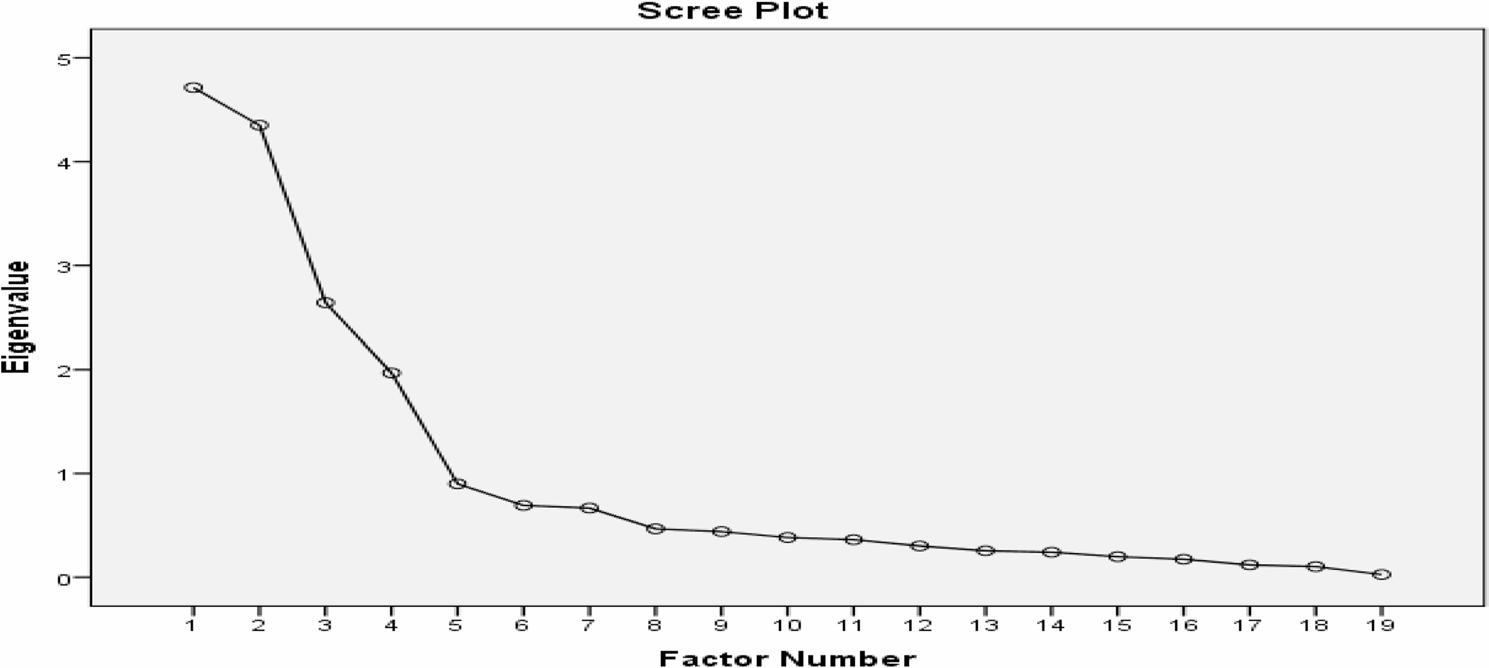
Scree plot for factor identification

To examine and assess the number of factors that explained the majority of the variance in the WLB scale, the scree plot was also used. In this process, all factors with Eigenvalues greater than 1 (one) was retained. Thus, for further analysis strategies maximum likelihood method with promax rotation method four factors with Eigenvalues > 1 were observed on the scree plot. Figure 1, depicted the scree plot of the 19-items of work-life balance.

### Confirmatory factor analysis results of WLB scale

The recommendations given on the model fit indices in the maximum likelihood (ML) models by researchers such as [40–42] were distinguish between types of fit indices as absolute fit indices, relative (or comparative) fit indices, and the non-centrality-based Indices for evaluating a given model fit. Absolute Fit Indices includes chi-square values (χ^2^ = < 3 >1); goodness-of-fit index (GFI *≥* 0.90); and the standardized root mean square residual (SRMR = < 0.08). The relative fit indices includes Tucker-Lewis Index (TLI >0.90); Normed Fit Index (NFI >0.95), and Incremental Fit Index (IFI >0.90). The non-centrality-based Indices includes root mean squared error of approximation (RMSEA = < 0.08), relative non-centrality Index (RNI >0.90); and comparative fit index (CFI >0.90).

However [40], have proposed the range of the cut-values of the fit indices for RMSEA (< 0.08), for CF (>0.90), for TLI (>0.90), for NFI (>0.90), for GFI (>0.90), and for SRMR (< 0.08) as acceptable for a model fit [41]. Found that TLI, CFI, and RMSEA tend to be too conservative in selecting a given models. Table 8, presented the first order of CFA models of the WLB scale.

**Table 8:** Fit indices for CFA models of the four subscales of the WLB scale

Subscale	χ2	GFI	CFI	TLI	RMSEA	SRMR
WEPL	2.048	0.998	0.999	0.990	0.056	0.0078
PLEW	2.272	0.992	0.996	0.988	0.061	0.0104
WIPL	2.301	0.976	0.990	0.938	0.073	0.0080
PLIW	0.285	0.999	1.000	1.007	0.000	0.0057
Recommended Value	< 3 > 1	> 0.90	> 0.90	> 0.90	< 0.08	< 0.06

*χ2* Chi-square, *GFI* Goodness of Fit Index, *TLI* Tucker-Lewis Index, *CFI* Comparative Fit Index, *RMSEA* Root Mean Square Error of Approximation, *SRMR* Standardized Root Mean Squared Residual

As shown in Table 8, the results of first order CFA revealed that all the four dimensions of WLB scale fit the data well in line with the recommended values of fit indices by the researchers [36, 40–42]. The fit of measurement model using the criteria mentioned by the researchers which affirmed WLB as four dimension construct that consisted of PLEW, WEPL, PLIW, and WIPL. The correlated four-factor solution measurement model with standardized estimates for work-life balance of health professionals derived using CFA analysis. All indicators were reflective which measured the four factor WLB dimensions. The measurement error (e) of each indicator represents the unique variances which were not explained by the unobserved variables. However, as CFA results indicated, the initial hypothesized model did not fit the data well. Based on the guide gained from modification indices correlating *(e-2 & e-3*,* e-7 & e-8*,* and e-16 & e-17)*,* we* created better model specification for WLB measurement scale.

Finally the model fit the data well: χ2 = 414.629 (df = 142, *p* <.000), χ2/df = 2.920, TLI = 0.934, CFI = 0.945, RMSEA = 0.075(90% CI: 0.067–0.084), and SRMR = 0.0655.

The measurement model for WLB had 190 distinct sample moments with 48 estimated distinct parameters which resulted in 142 (190 − 48) degrees of freedom, indicating an over identified model. The model consisted of 42 variables; of which 23 were (Exogenous) unobserved variables and 19 were (Endogenous) observed variables.

The results of CFA indicated that the model has a total of 38 regression weights, 23 of which were fixed and 15 were freely estimated. It also consisted of 10 covariances and 23 variances, all of which were estimated. In general, the final measurement model for work-life balance of health professionals had a total of 71 parameters, 48 of which were estimated and 23 were fixed. Table 9 summarizes the model fit indices of work-life balance scale.

**Table 9:** Results of goodness-of-fit test for work-life balance scale

Model Fit Indices	χ2	GFI	TLI	CFI	RMSEA	SRMR
Model Value	2.920	0.919	0.934	0.945	0.075	0.0655
Recommended Value	< 3.0	> 0.90	> 0.90	> 0.90	< 0.08	< 0.06

*χ2* Chi-square, *GFI* Goodness of Fit Index, *TLI* Tucker-Lewis Index, *CFI* Comparative Fit Index, *RMSEA* Root Mean Square Error of Approximation, *SRMR* Standardized Root Mean Squared Residual

**Fig. 2:**
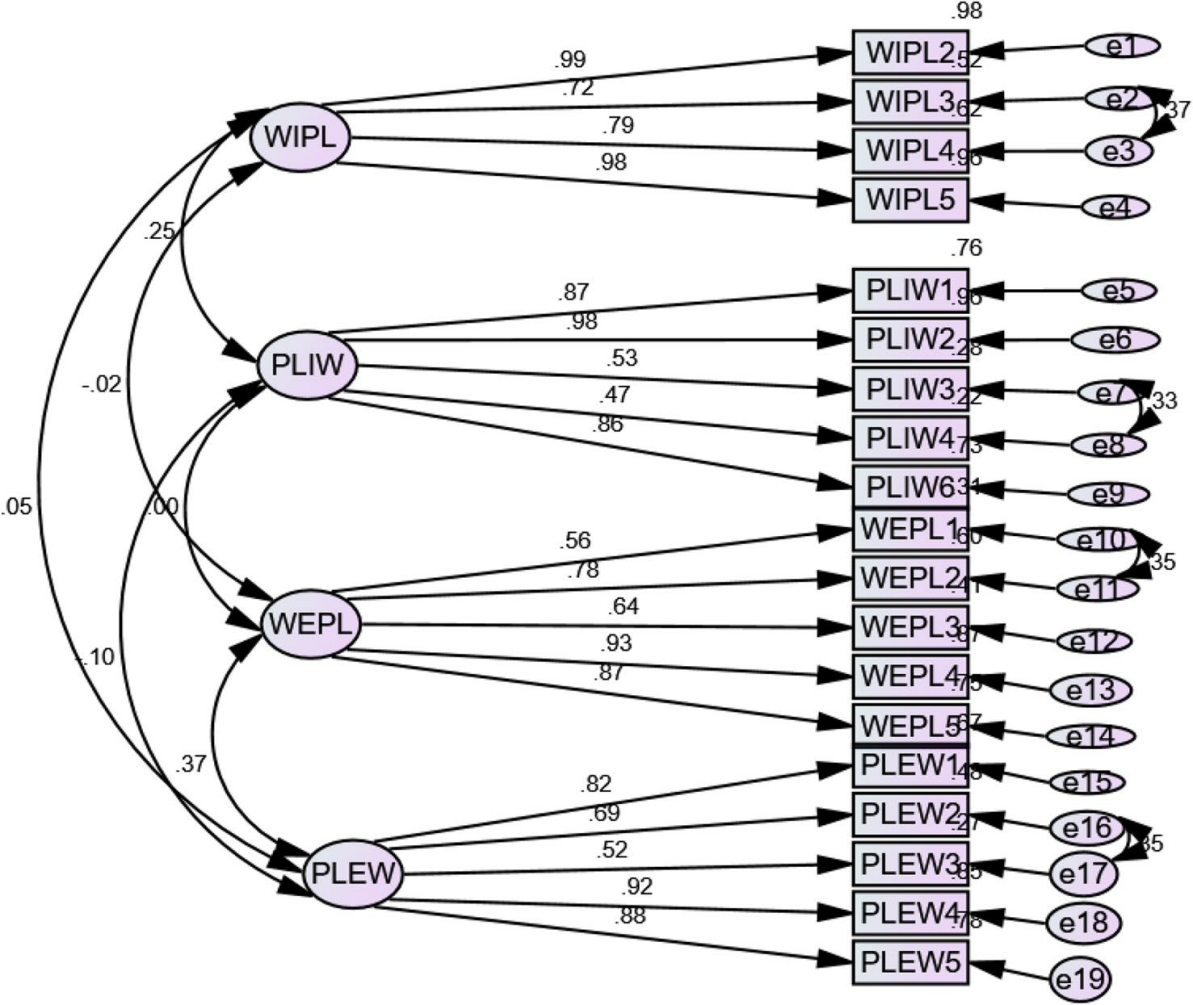
Measurement model for health professional’s work-life balance scale

As shown in Table 9, the results of CFA indicated that the four factor model, x^2^ (142, *N* = 338) = 414.628, GFI = 0.919, TLI = 0.934, CFI = 0.945, RMSEA = 0.075, and SRMR = 0.0655 were fit the data better [41]. forwarded the comprehensive evaluations of cutoff criteria that suggested the good fit between the target model and the observed data (assuming maximum likelihood model estimation) is obtained in instances where SRMR values are close to 0.08 or below; RMSEA values are close to 0.08 or below; and CFI and TLI values are close to 0.90 or greater. Figure 2 presents the final measurement model of the work-life balance scale.

## Discussions

This study attempted to examine the measurement model structure of work-life balance of health professionals in Eastern Ethiopia. Based on the review of literature, health professionals’ work-life balance was hypothesized as a four-factor construct consisting of work interference with personal life, personal life interference with work, work enhancement of personal life, and personal life enhancement of work as core domains.

Accordingly, CFA results indicated that the work-life balance is a latent construct comprising four major dimensions, namely work interference with personal life, personal life interference with work, work enhancement of personal life, and personal life enhancement of work as core domains. The result of this study was in line with the theoretical model that describes work-life balance as an integration of work interference with personal life, personal life interference with work, work enhancement of personal life, and personal life enhancement of work [14–17]. Likewise, as hypothesized, this study demonstrated work-life balance as a multidimensional construct comprising four sub-dimensions: PLEW, WEPL, PLIW, and WIPL. This finding lends support to evidence documented in comprehensive reviews of the dimensions of work-life balance [19, 20]. The newly adapted and validated health professionals’ work-life balance scale was found to be a four-factor subscale such as PLEW, WEPL, PLIW, and WIPL. The final WLB scale has shown good reliability estimates, model fit, discriminant validity, and convergent validity. Thus, it would suffice to say that the WLB scale measures what it purports to measure.

Based on the results from EFA and CFA, this study approved the 19-item WLB scale as appropriate for measuring the work-life balance of health professionals. The Cronbach alpha coefficients for the four dimensions reported by [19] were for the WIPL subscale (0.91), the PLIW subscale (0.85), the WEPL subscale (0.75), and the PLEW subscale (0.90). However, the current study reported that the Cronbach alpha coefficient for WLB dimensions was the overall composite reliability ranging between (0.88) for WEPL, (0.86) for PLIW, (0.88 for PLEW, and (0.93) for WIPL, which indicated that WIPL and PLIW were similar to the Cronbach alpha coefficient reported by [19].

According to [28] reliability is defined as the consistency or repeatability of an instrument. The most important form of reliability for multi-item instruments is the instrument’s internal consistency, which is the degree to which sets of items on an instrument behave in the same way. A scale’s internal consistency is quantified by a Cronbach’s alpha value that ranges between 0 and 1, with optimal values ranging between 0.7 and 0.9 [28].

The purpose of this study was to investigate the psychometric properties and examine the factor and structure analysis of WLB scale among health professionals in public and private hospitals in Eastern Ethiopia. Therefore, in the present study, WLB dimensions namely (WIPL, PLIW, WEPL, and PLEW) described as the four factor dimensions of WLB scale. Thus, this study used a cross-sectional design to examine the validity, reliability, factor structures, and psychometric properties of WLB scale for health professionals in public and private hospitals in Eastern Ethiopia.

In this study, before conducting the process of data analysis, the underlying assumptions for EFA and CFA were checked. CFA was conducted to investigate how well a four-factor WLB measurement model, which was identified in this study with EFA, fits the new sample data. Therefore, the fit between the model and the data was evaluated using the chi-square test; the fit indices such as GFI, RMSEA, SRMR, TLI, CFI, and the chi-square/df ratio; and significance tests were checked for the WLB scale.

Various studies have been conducted concerning the WLB scale for employees in educational settings, bank and insurance sectors, and manufacturing industries. The cross-cultural adaptation and validation of WLB scale were attempted across various nations worldwide. Researchers have been developed various level dimensions of WLB assessment tools, including the one dimensional and multidimensional constructs of WLB. For instance [43], developed and validated WLB scale as one dimensional which consists of 8-items with the Cronbach’s alpha value of 0.92. Their study indicated that the WLB scale was valid and reliable for a Turkish employee sample. On the other hand [44], developed and validated WLB scale as one dimensional which consists of 4-items with the Cronbach’s alpha value of 0.93 for Australia and 0.84 for New Zealand samples respectively. However, the collective culture context of Ethiopia might be different from Western nations such as Australia and New Zealand and required more items to measure WLB of health professionals in our socio-cultural contexts.

 Agha et al. [45] conducted development and validation of WLB scale in Oman and found that WLB scale has three dimensions which consists of 15-items namely WIPL with 7-items, PLIW with 4-items, and WEPL 4-items. They reported the three subscales has Cronbach’s alpha of 0.9, 0.9, and 0.8 respectively.

Hayman [15] conducted the psychometric assessment of WLB on administrative and professional employees from a large university in Western Australia and found that WLB scale has three dimensions which consists of 16-items namely WIPL with 8-items, PLIW with 4-items, and WPLE with 4-items. They reported the final Cronbach alpha values for the three factors include 0.93 for WIPL, 0.85 for PLIW, and 0.69 for WPLE. As we can see from the results of WLB scale, the higher reliability values were reported in Oman than the study done in Australia. Therefore, possible to say that there are cross-cultural variations of psychometric properties of WLB scale. As such, it is vital to examine psychometric properties of WLB scale of Amharic version in Ethiopian context.

 Smeltzer et al. [16] investigated the psychometric properties of the WLB scale among nurse faculty involved in doctoral education in America and found that WLB scale has three dimensions which consists of 15-items namely WIPL with 7-items, PLIW with 4-items, and WPLE with 4-items.They reported the Cronbach’s alpha coefficients for reliability of the scale were 0.88 for the total scale and for the subscales were 0.93 for WIPL, 0.85 for PLIW, and 0.69 for WEPL. Even though the Cronbach’s alpha coefficients for WIPL and PLIW reported by [16] were similar with the current study, the Cronbach’s alpha coefficients for WEPL was low which is below the cut-point of (0.75).

Thus, considering the multidimensionality of WLB measure, the current study prefer the more robust and standardized scale of WLB by [19] which previously published by the American psychology Association. According to [19], WLB measure perceptions of the extent to which work interferes with personal life and personal life interferes with work, and the extent to which work enhances personal life and personal life enhances work.

In this study, the calculated reliability for each of the four WLB subscales shows that all had acceptable internal consistency which ranges from 0.884 to 0.933. In this study, WIPL subscale had high internal consistency of 0.933 Cronbach’s alpha coefficient but WEPL and PLEW had 0.884 Cronbach’s alpha coefficient which is low compared to other two components. Consistent with this study [19], reported the same reliabilities with the WIPL and PLIW subscale in the American contexts. A study by [15] revealed that the Cronbach alpha values for the WLB scale which include 0.93 for WIPL, 0.85 for PLIW, and 0.69 for WEPL for professional employees in Western Australia. Both [15, 19] reported lower Cronbach alpha values for the WEPL and PLEW subscales when compared to the current study. Probably, the new items added on these two dimensions might create differences because of sociocultural variation among these nations.

Health professionals are always striving hard to save lives, as they are front-liners in dealing with patient care services. The study conducted by [46] yielded that maintaining nurses’ WLB is critical to improving healthcare productivity and the delivery of quality patient care. According to [47], health professionals who manage to maintain a balance between their work and personal lives are less likely to experience burnout and more likely to report higher job satisfaction and better mental health. Moreover [48], argued that individuals who lack WLB have more work and home commitments, work longer hours, and lack personal time. To date, the limited amount of cross-cultural research on the WLB issues among health professionals is also evidenced in Ethiopian context. Therefore, the current study sought to expand the body of knowledge and fulfill the gaps and limitations regarding the cross-cultural validation of WLB scale from the Western context to our local socio-cultural context.

The previous findings were replicated WLB scale in the Maltese and Italian context [49]. In both groups, the CFA measurement models showed better goodness-of-fit indices. However, the collective culture context of Ethiopia might be different from Western nations such as America, Australia, Italia, Malta, and New Zealand context and required more items to measure WLB of health professionals in our socio-cultural contexts. Therefore, in this study context, four items were added on the 17-items developed by [19]. Among a total of 21-items of WLB scale, 19-items were validated and standardized in the current study.

The psychometric property assessment was ensured through the following three major processes, namely factor analysis, reliability analysis, and validity analysis, which includes EFA, CFA, Cronbach’s alpha and composite reliability, content, convergent and divergent validity ensuring the unidimensionality explains whether all items are measuring each theoretical variable or construct. Multiple goodness-of-fit index had used to test the Amharic version of WLB scale for health professionals in Eastern Ethiopian context. Thus, the goodness-of-fit indices is calculated through CFA and the main measures for goodness-of-fit are explained through the GFI, CFI, TLI, RMSEA, and SRMR cut-of-criteria have been ensured.

The results of the current study indicated that CFA of all the four dimensions of WLB scale fit the data well in line with the recommended values of fit indices by the researchers [e.g. 36, 40–42], which are the most cited cutt-of-criteria in most literature. The fit of measurement model using the criteria mentioned by the researchers which affirmed WLB as four dimension construct that consisted of PLEW, WEPL, PLIW, and WIPL. The issues of reliability and validity test were run with the help of Cronbach’s alpha, composite reliability, content validity, convergent and divergent validity which is indicated as the Amharic version WLB scale for measuring the WLB of physicians, nurses, and midwives had excellent reliability and validity characteristics.

The cultural aspects affecting WLB in Ethiopian healthcare professionals might related to the Ethiopian healthcare system, which characterized by limited resources and a critical shortage of human resources specially in public hospitals. In this challenging context, the concepts of work-life balance among health professionals might influenced by the work, family, and personal lives which in turn affects the overall effectiveness of the health system. A study in public hospitals found that a significant majority of physicians reported their work schedule negatively affecting their family life [50]. Moreover, the study in Ethiopian context revealed that the combination of night shifts, high-stakes emergencies, and the emotional labor involved contributes to a significant work-life imbalance, which is a key factor cited in their intention to leave the profession [51]. When health professionals are unable to balance their professional and personal lives, it leads to work-life conflict or work-family conflict because of high stress levels and burnout.

Addressing the issues of WLB among health professionals requires critical investigations by researchers, public health institutions, policymakers, and hospital administrators, focusing not only on financial incentives but also on improving the work, family, and personal lives that enable a healthier and good balance between the professional and personal lives of health professionals.

Following EFA and CFA analysis, the findings of the study indicated that the better model specification for the WLB measurement scale fit the observed data well: *x*^2^ = 414.628 (df = 142, *p* <.001), *x*^2^/df = 2.920, GFI = 0.919, TLI = 0.934, CFI = 0.945, RMSEA = 0.075, and SRMR = 0.0655. Therefore, as per the reliability and validity test, the measurement model shows that work-life balance tool possess all reliability and validity required for standardizing the tools for health professionals in public and private hospitals in Eastern Ethiopia.

## Conclusions

Health professionals are the backbone of the healthcare sectors. The balance between work and non-work affairs has been a growing issue globally among health professionals. In fact, balancing work and non-work affairs are the most challenging thing for health professionals because of their multiple duties and responsibilities related to their work, family, and personal lives. Nevertheless, very few studies have reported the issues of work-life balance among health professionals in health care settings in Ethiopia.

This work-life balance tool would be particularly useful in health care settings where health professionals such as physician, nurse, and midwife directly interact with their patients. This tool attempted to examine the factors and structure of WLB that can be considered significant. This tool helps to measure the four dimensions of work-life balance. By checking the level of work-life balance of health professionals, this tool helps measure the levels of interference and enhancement in their inner strength to increase the level of balancing between work and personal life.

There is a need for adapting and validating work-life balance scale that would reflect the current work-life balance measure among health professionals in public and private hospitals in Eastern Ethiopia. Therefore, the first task of this study was to adapt, validate, and translate the English version of WLB scale in to the Amharic version in the context of Eastern Ethiopia for health professionals. This study confirmed that the WLB scale consists of 19 items grouped into four factors, among which four were newly developed items based on the responses of 338 health professionals selected from both public and private hospitals.

The Ethiopian/Amharic version of the WLB scale can be used as reliable instruments to assess work-life balance of health professionals in public and private hospitals in an Eastern Ethiopian context. The WLB questionnaires were four-dimensional, like the original questionnaires developed by [19]. This instrument can be easily applied to evaluate the work-life balance of health professionals. Thus, the Ethiopian versions of WLB scale are reliable and valid measures to assess work-life balance among health professionals in public and private hospitals in Ethiopia.

As per the reliability and validity test of the current study, it shows that work-life balance tool possess all reliability and validity required for standardizing tool. This tool will help to check the level of work-life balance among health professionals working in public and private hospitals in Eastern Ethiopia. This particular tool can be used by the public and private hospitals in Ethiopia. and related sectors for their study purpose.

### Limitations

Although this study provides relevant and previously undocumented findings, it has some limitations that would be considered for future studies. First, the current study used self-reported surveys, health professionals’ may have overestimated or underestimated their levels of WLB which could lead to response bias. Second, the newly adapted, translated, and validated instrument designed to measure WLB among workers in large company of Western context. However, the job status, role, and titles of health professionals may differ from the large industrial workers. Third, this study was limited to government public hospitals and private hospitals in Dire Dawa City Administration and Harari Regional State in Eastern Ethiopia. Thus, the findings cannot be generalized to clinics, health centers, health posts, communities, and missionary hospitals. Therefore, future research could move some steps forward to assess the psychometric properties of the instrument by including large samples across the country. Fourth, this study was limited to cross-sectional design with quantitative research approach. However, longitudinal design and qualitative cognitive interview will need to prove the WLB measure in cross-cultural contexts.

## Data Availability

The data that support the findings of this study are available upon request from the corresponding author.

## References

[1] Guest DE. Perspectives on the study of Work-life balance. Social Sci Inform. 2002;41(255):255–79. 10.1177/0539018402041002005.

[2] Allen TD. The Work–Family Role Interface: A Synthesis of the Research from Industrial and Organizational Psychology. In Irving B. Weiner, editors, Handbook of Psychology. John Wiley & Sons, Inc. 2013;699–705

[3] Greenhaus JH, Beutell NJ. Sources of conflict between work and family roles. Acad Manage Rev. 1985;10(1):76–88.

[4] Frone MR, Russell M, Cooper ML. Antecendents and outcomes of Work-Family conflict: testing A model of Work-Family interface. J Appl Psychol. 1992;77(1):65–78. 1556042 10.1037/0021-9010.77.1.65

[5] Adams JA, King LA, King DW. Relationships of job and family Involvement, family social Support, and Work-Family conflict with job and life satisfaction. J Appl Psychol. 1996;81(4):411–20.

[6] Amstad FT, Meier LL, Fasel U, Elfering A, Semmer NK. A Meta-Analysis of Work–Family conflict and various outcomes with a special emphasis on Cross-Domain versus Matching-Domain relations. J Occup Health Psychol. 2011;16(2):151–69. 10.1037/a0022170. 21280939 10.1037/a0022170

[7] Baltes BB, Clark MA, Chakrabarti M. Work-Life Balance: The Roles of Work-Family Conflict and Work-Family Facilitation. In: Oxford Handbook of Positive Psychology and Work. Oxford University Press; 2015.

[8] Wilson KS, Baumann HM. Capturing a more complete view of employees’ lives outside of work: the introduction and development of new interrole conflict constructs. Pers Psychol. 2015;68:235–82. 10.1111/peps.12080.

[9] Soomro A, Breitenecker R, Shah S. Relation of Work-life balance, Work-family conflict, and Family-work conflict with the employee performance: moderating role of job satisfaction. South Asian J Bus Stud. 2018;7(1):129–46. 10.1108/SAJBS-02-2017-0018.

[10] Venkatesan R. Measuring work-life balance: relationships with work-family conflict and family-work conflict. J Strategic Hum Resource Manage. 2021;10(2):28–36. http://publishingindia.com/jshrm/.

[11] Kalliath T, Brough P. Work–life balance: a review of the meaning of the balance construct. J Manage Organ. 2008;14(3):323–7.

[12] Parkes LP, Langford PH. Work–life balance or work–life alignment? A test of the importance of work–life balance for employee engagement and intention to stay in organisations. J Manage Organ. 2008;14(3):267–84.

[13] Matsuo M, Suzuki E, Takayama Y, Shibata S, Sato K. Influence of striving for work–life balance and sense of coherence on intention to leave among nurses: a 6-month prospective survey. J Health Care Organ Provis Financ. 2021;58:1–9. 10.1177/00469580211005192. 10.1177/00469580211005192 PMC8743965 33769128

[14] Fisher-McAuley G, Stanton J, Jolton J, Gavin J. Modeling the Relationship between Work Life Balance and Organizational Outcomes. Proceedings of the Annual Conference of the Society for Industrial-Organizational Psychology. 2003; 1–26. Orlando, FL, USA.

[15] Hayman J. Psychometric assessment of an instrument designed to measure work life balance. Res Pract Hum Resource Manage. 2005;13(1):85–91.

[16] Smeltzer S, Cantrell M, Sharts-Hopko N, Heverly M, Jenkinson A, Nthenge S. Psychometric analysis of the work-life balance self-assessment scale. J Nurs Meas. 2016;24(1):5–12. 10.1891/1061-3749.24.1.5. 27103238 10.1891/1061-3749.24.1.5

[17] Smith J, Ryan L, Sonnega A, Weir D. Psychosocial and lifestyle questionnaire 2006–2016. Ann Arbor, Michigan: Survey Research Center Institute for Social Research University of Michigan; 2017.

[18] Boakye AN, Asravor RK, Essuman J. Work-life balance as predictors of job satisfaction in the tertiary educational sector. Cogent Bus Manage. 2023;10(1):2162686. 10.1080/23311975.2022.2162686.

[19] Fisher GG, Bulger CA, Smith CS. Beyond work and family: a measure of work/Nonwork interference and enhancement. J Occup Health Psychol. 2009;14(4):441–56. 10.1037/a0016737. 19839663 10.1037/a0016737

[20] Hsieh YJ, Pearson TE, Kline SF. The moderating effects of job and personal life involvement on the relationship between work-personal life conflict and intention to quit. J Hum Resour Hosp Tour. 2009;8:1–14. 10.1080/15332840802274387.

[21] Haar JM, Russo M, Suñe A, Ollier-Malaterre A. Outcomes of work–life balance on job satisfaction, life satisfaction and mental health: a study across seven cultures. J Vocat Behav. 2014;85:361–73.

[22] Avadhani VD, Menon RB. Development and standardization of the work-life balance scale for the insurance sector employees. Cogent Bus Manage. 2022;9(1):2154994. 10.1080/23311975.2022.2154994.

[23] Kothari CR. Research methodology: methods and techniques. 2nd ed. New Delhi: Wishwa Prakashan; 2004.

[24] Singh AS, Masuku MB. Sampling techniques & determination of sample size in applied statistics research: an overview. Int J Econ Commer Manag. 2014;2(11):1–23.

[25] Yamane T, Statistics. An introductory analysis. 2nd ed. New York: Harper and Row; 1967.

[26] Lawshe C. A quantitative approach to content validity. Pers Psychol. 1975;28:563–75.

[27] Beaton D, Bombardier C, Guillemin F, Ferraz MB. Guidelines for the process of cross-cultural adaptation of self-report measures. Spine. 2000;25(24):3186–91. 11124735 10.1097/00007632-200012150-00014

[28] Creswell JW, Creswell JD. Research design: Qualitative, quantitative, and mixed methods approaches. 5th ed. United States of America: SAGE Publications, Inc.; 2018.

[29] Petscher Y, Schatschneider C, Compton DL. Applied Quantitative Analysis in Education and the Social Sciences. New York: Routledge, Taylor & Francis; 2013.

[30] Watkins MW. A step-by-step guide to exploratory factor analysis with SPSS. Taylor & Francis Group, New York, USA: Routledge; 2021.

[31] Shrotryia VK, Dhanda U. Content validity of assessment instrument for employee engagement. SAGE Open. 2019;1–7. 10.1177/2158244018821751.

[32] Yusoff MSB. ABC of content validation and content validity index calculation. Educ Med J. 2019;11(2):49–54. 10.21315/eimj2019.11.2.6.

[33] Kurbi HA, Abebe SM, Mengistu NW, Ayele TA, Toni AT. Cultural adaptation and validation of the amharic version of the world health organization’s self reporting questionnaire (SRQ-20) screening tool among pregnant women in North West ethiopia: a psychometric validation. Int J Womens Health. 2023;18(15):779–91. 10.2147/IJWH.S402865. 10.2147/IJWH.S402865 PMC10202208 37223068

[34] Polit DF, Beck CT, Owen SV. Focus on research methods is the CVI an acceptable indicator of content validity? Appraisal and recommendations. Res Nurs Health. 2007;30:459–67. 10.1002/nur.20199. 17654487 10.1002/nur.20199

[35] Kline RB. Principles and practice of structural equation modeling. 4th ed. New York, NY: The Guilford Press; 2016.

[36] Hair JF, Black WC, Babin BJ, Anderson RE. Multivariate data analysis. 8th ed. United Kingdom: Cengage Learning, EMEA; 2019.

[37] Collier JE. Applied structural equation modeling using AMOS: basic to advanced techniques. New York, NY: Routledge; 2020.

[38] Pallant J. SPSS, SURVIVAL MANUAL: A step by step guide to data analysis using SPSS. 4th ed. Australia: Published by Allen & Unwin Book Publishers; 2010.

[39] Brown TA. Confirmatory factor analysis for applied research. 2nd ed. New York, NY: The Guilford Press; 2006.

[40] Hu LT, Bentler PM. Fit indices in covariance structure modeling: sensitivity to underparameterized model misspecification. Psychol Methods. 1998;3:424–53.

[41] Hu LT, Bentler PM. Cutoff criteria for fit indexes in covariance structure analysis: conventional criteria versus new alternatives. Struct Equ Model. 1999;6:1–55.

[42] Yuan KH, Chan W, Marcoulides GA, Bentler PM. Assessing structural equation models by equivalence testing with adjusted fit indexes. Struct Equ Model. 2016;23(3):319–30.

[43] Tasdelen-Karckay A, Bakalım O. The mediating effect of work–life balance on the relationship between work–family conflict and life satisfaction. Aust J Career Dev. 2017;26(1):3–13. 10.1177/1038416216682954.

[44] Brough P, Timms C, Chan XW, Hawkes A, Rasmussen L. Handbook of Socioeconomic Determinants of Occupational Health, Handbook Series in Occupational Health Sciences. Switzerland: Springer Nature; 2020.

[45] Agha K, Azmi FT, Khan SA. Work-Life balance: scale development and validation. In: Heras ML, Chinchilla N, Grau M, editors. The Work-Family balance in light of globalization and technology. Newcastle upon Tyne, UK: Cambridge Scholars Publishing; 2017. pp. 109–30.

[46] Rony MK, Numan S, Alamgir S. The association between work-life imbalance, employees’ unhappiness, work’s impact on family, and family impacts on work among nurses: a cross-sectional study. Inform Med Unlocked. 2023;38:101226. 10.1016/j.imu.2023.101226.

[47] Zhang J, Rehman S, Addas A, Ahmad J. Influence of work-life balance on mental health among nurses: the mediating role of psychological capital and job satisfaction. Psychol Res Behav Manage. 2024;17(1):4249–62. 10.2147/PRBM.S497305. 10.2147/PRBM.S497305 PMC11646404 39679316

[48] Alotaibi A, Aldossry T. Work-life balance of working mothers in the healthcare industry in Riyadh, Saudi Arabia. J Soc Serv Res. 2024;50(1):14–24. 10.1080/01488376.2023.2271037.

[49] Bottaro R, Giovanni KD, Faraci P. Assessing work–life balance in Malta and Italy: a cross-cultural investigation using exploratory structural equation modelling (ESEM). Psychol Res Behav Manag. 2025. 10.2147/PRBM.S529101. 40766172 10.2147/PRBM.S529101 PMC12323781

[50] Ayalew E, Worku A, Mosisa G. Work-life balance and associated factors among physicians in public hospitals of West Ethiopia. BMC Health Serv Res. 2019;19(1):1–8.30606168

[51] Tsegaye S, et al. Intention to leave and associated factors among midwives working in public health institutions of addis Ababa, Ethiopia. BMC Pregnancy Childbirth. 2020;20(1):1–10. 10.1186/s12884-020-2734-4 PMC6975085 31964349

